# Wearable Sensors for the Assessment of Functional Outcome Following Reverse Shoulder Arthroplasty: A Systematic Scoping Review

**DOI:** 10.3390/jcm14186401

**Published:** 2025-09-10

**Authors:** Peter K. Edwards, Jay R. Ebert, William G. Blakeney, Stefan Bauer, Allan W. Wang

**Affiliations:** 1 School of Allied Health, Curtin University, Perth, WA 6102, Australia; 2 School of Human Sciences, University of Western Australia, 35 Stirling Hwy, Crawley, WA 6009, Australia; 3 Royal Perth Hospital, Perth, WA 6000, Australia; 4 Department of Surgery, University of Western Australia, 35 Stirling Hwy, Crawley, WA 6009, Australia; 5 Shoulder Surgery and Upper Limb Center, Ensemble Hospitalier de La Côte, 1110 Morges, Switzerland

**Keywords:** reverse shoulder arthroplasty, function, movement, technology, inertial measurement units

## Abstract

This scoping review assessed the current use of wearable sensors in monitoring recovery following reverse shoulder arthroplasty (RSA). A systematic search of electronic databases was undertaken (MEDLINE, EMBASE, CINAHL, and Web of Science) between 2005 and 2024 following the PRISMA-ScR protocol. Studies were eligible if they were peer reviewed, available in full text, and reported the use of wearable sensors to evaluate shoulder motion or activity in postoperative RSA patients. Fifty-seven studies were identified, of which six met the inclusion criteria. Studies were either focused on assessing shoulder motion (n = 3) or on measuring upper limb activity counts or activity intensities (n = 3); however the calculation of output variables were different across most studies. Sensors were positioned on the operated upper arm in all studies, though sensor placement on the sternum and the wrist varied. Session durations ranged from 24 h to continuous monitoring beyond seven days. Daily wear times were most commonly during full waking hours. The large variation in wearable sensor configuration, testing protocols, and the calculation of output variables limited the comparability across studies. Standardization in sensor protocols and outcomes is required to enable the reliable wearable assessment of postoperative recovery after RSA.

## 1. Introduction

Reverse shoulder arthroplasty (RSA) is common and now accounts for over two-thirds of all shoulder replacement procedures [1]. RSA, traditionally, has been recommended for older patients with low activity demands, though indications now include younger patients with higher activity demands, such as sports and recreational activities [2,3,4]. Assessing the recovery of upper limb function following RSA requires outcome measures that can not only detect meaningful changes in patient function, but are also reflective of a patient’s normal function. Patient-reported outcome measures (PROMs) are commonly used to evaluate postoperative pain and function, though are limited by potential recall bias and ceiling effects, most of which are common in high-functioning patients [5,6,7]. Objective assessments such as goniometric range of motion and isometric strength testing are good alternatives to PROMs. However, these are often performed only at a single point in time and may not accurately reflect how patients move or function outside of a clinical setting.

Wearable sensor technology is now used in orthopedic settings to continuously monitor the movement-related outcomes of patients in their own environments [8]. Wearable sensors include accelerometers that have the ability to record linear accelerations across one, two, or three axes to quantify limb or whole-body movement. Inertial measurement units (IMUs) are more advanced sensors that integrate accelerometers with other sensors, such as gyroscopes and/or magnetometers, to capture more detailed, multi-directional data on limb motion and orientation relative to the body’s position and movement in space. For assessing outcomes following RSA, wearable sensors are an alternative to PROMs and traditional objective tests in clinics by capturing upper limb activity and/or arm motion, potentially providing a more detailed and ecologically valid assessment of the functional capacity and recovery of patients.

Despite their widespread adoption in consumer fitness and increasing use in lower limb arthroplasty [8,9,10], wearable sensors appear to be underexplored in the shoulder, in particular in shoulder arthroplasty. It is not clear as to how wearable sensors, in particular methodologies such as sensor placement, wear time, duration of monitoring, and outcome metrics, are being applied. This systematic scoping review aims to assess the current methodology and clinical application of wearable sensor technology to evaluate functional outcomes following RSA.

## 2. Materials and Methods

We conducted a scoping review in accordance with the Joanna Briggs Institute Methodology for JBI Scoping Reviews [11] and adhered to the PRISMA Extension for Scoping Reviews (PRISMA-ScR) checklist for reporting [12], though the review was not registered.

### 2.1. Data Sources and Search Strategy

Four electronic databases (MEDLINE, EMBASE, Web of Science, CINAHL) were searched from January 2005 up to and including August 2024. An example of a search strategy used is provided in Table 1.

### 2.2. Study Selection

Studies were eligible if they reported using wearable sensors for the purpose of monitoring or assessing patient function, activity, or mobility following RSA. Studies assessing this in clinical or home-based settings were included. No study was excluded based on the diagnostic indication for RSA (e.g., osteoarthritis, cuff tear arthropathy). We included a range of study designs, including cohort studies, cross-sectional studies, and intervention studies, as long as wearable sensor-derived data was reported in the context of RSA recovery and postoperative outcome. Any cadaveric or animal studies, case reports, protocol-only publications without postoperative data, and non-peer-reviewed articles were also excluded. Systematic reviews were not included in the final analysis, but their reference lists were hand-searched for additional eligible studies.

On completion of the comprehensive searches on each database, studies were exported to Covidence systematic review software (Veritas Health Innovation, Melbourne, Australia. Available at www.covidence.org). Duplicates were removed first by Covidence and second by manual verification. Following this, the screening of manuscript titles and abstracts was undertaken by two authors (P.E. and J.E.). Following title and abstract review, the same two authors evaluated the subsequent full-text manuscripts for inclusion in the final review. Any disagreements following full- text assessment were discussed between reviewers followed by a consensus.

### 2.3. Data Extraction, Synthesis, and Analysis

Data from all included full-text manuscripts were extracted. Details from these studies were entered into tables and cross-checked by two authors (P.E and J.E). Study characteristics included the study design, sample size (including arthroplasty and control groups, if relevant), follow-up time points, the setting in which data was collected, and the type of RSA implant used. The details of the wearable sensors and protocols included sensor type (e.g., accelerometers, gyroscopes, magnetometers, GPS), number of axes measured, sampling frequency, number of sensors used in the evaluation, sensor placement, and information regarding the session length and wear time whereby data was collected. Finally, wearable sensor outcome variables were documented to summarize the category (or scope) in which the wearable sensors were used (i.e., to collect information on motion or activity) as well as metrics reported in each study. Quality assessment was not performed.

## 3. Results

**Figure 1:**
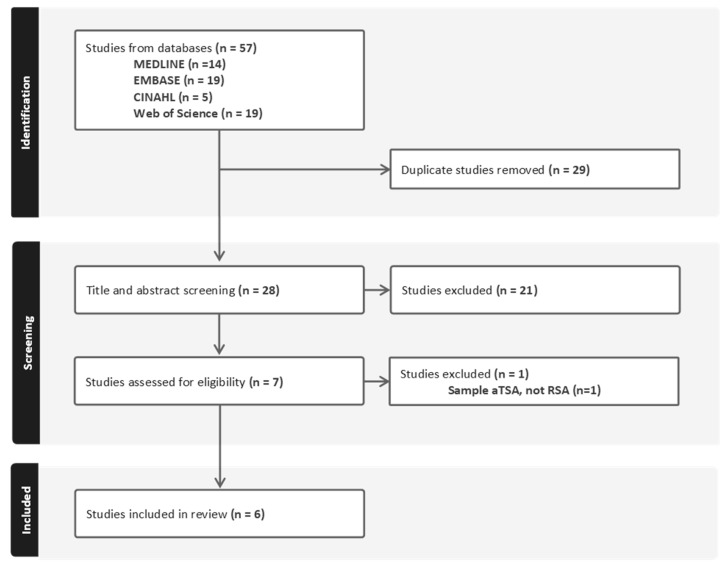
Preferred Reporting Items for Systematic Reviews and Meta-Analyses extension for Scoping Reviews (PRISMA-ScR) flow chart.

The search strategy produced 57 results, including 14 from MEDLINE, 19 from EMBASE, 5 from CINAHL, and 19 from Web of Science (Figure 1). After the removal of 29 duplicates, 28 titles and abstracts were screened, of which 7 were assessed as full texts (Figure 1). Of these, six studies were included in the review.

Study characteristics are summarized in Table 2. All six studies recruited older adults undergoing RSA, with follow-ups ranging from 24 weeks to 24 months (Table 2). Two studies also included a healthy control group for comparison [13,14]. Three studies focused on shoulder activity [14,15,16] and three studies examined shoulder motion [13,17,18]. Data was collected in free-living environments, and most studies used a prospective design. Sample sizes ranged from 10 to 64 participants. Three studies [15,17,18] included at least three postoperative follow-ups, while others were limited to two postoperative follow-ups [16], one postoperative follow-up [14], or a single postoperative assessment [13].

A range of wearable sensor types were employed across studies (Table 3). Triaxial accelerometers were the most commonly used, which were done so either in isolation or combination with other sensors. Four studies used a combination of accelerometers and gyroscopes [13,14,17,18], while two used a combination of triaxial accelerometers, gyroscopes, and magnetometers [14,17] and two used a combination of triaxial accelerometers, gyroscopes, and a compass [13,18]. Two studies used triaxial accelerometers in isolation [14,15,16]. No consistent sample frequency was reported, with three studies reporting a sampling rate of 100 Hz [14,15,16], one 10 Hz [18], while two did not report the sampling frequency [13,17]. For sensor placement, all studies positioned at least one sensor on the operative arm, typically at the mid humerus, while three studies also positioned a sensor on the sternum as a reference point to enable the recording of angular shoulder motion, relative to that point [13,17,18]. Two positioned sensors on both upper arms [13,15] to permit a contralateral arm comparison. Only three studies positioned sensors on the wrist [13,15,16], with two studies placing them bilaterally [13,15] and one unilaterally on the operated side [19]. Session durations ranged from less than 24 h [13,14,18] to weeks [17]. Three studies had daily wear times of 8 to 10 h [15,16,17], while three had 24 h continuous wear across the session duration [13,14,18].

Three studies used wearable sensors the for purpose of collecting and reporting outcomes related to shoulder range of motion (Table 4) [13,17,18]. These studies reported range of motion metrics such as angular bins, the frequency of elevation events, and time spent above specific range of motion thresholds. Chapman et al. [17] examined both average and maximum elevation range of motion, and also tracked the time spent within 15° bins below 90° and 45° bins above 90° elevation [17]. Van de Kleut et al. [18] grouped elevation events above 90° into low- (≤3/min), moderate- (4–9/min), and high- (≥10/min) intensity categories. Three studies used wearable sensors to record upper limb activity, or upper limb use, in daily life (Table 4) [14,15,16]. These studies reported activity using metrics such as activity counts per epoch (e.g., <28 counts per 15 s to define inactivity), vector magnitudes, and the classification of time spent in inactive, low, and high activity states (Table 4) [15,16].

## 4. Discussion

Wearable sensors are increasingly being used in orthopedics to objectively assess patient function in free-living environments. Their application has largely been used to assess gait and activity recovery following total knee arthroplasty [8,9,10]. It is unclear to what extent they have been used following shoulder surgery, in particular RSA. In total, six studies were identified, suggesting large variation in methods including sensor placement, wear time, session periods/lengths, and outcome measures of metrics used to report recovery. This highlights the need for research to standardize measurement set-up and the selection metrics used to evaluate functional recovery, whether that is of upper limb activity or motion.

Accelerometers were the most common sensor used across the six studies. All studies used inertial sensors though only a few used the full IMU system, which included gyroscopes and magnetometers. Most studies placed sensors on the upper arm and/or sternum, which makes sense given the cohort and the likely interest in quantifying the motion of the upper arm. Although, not placing sensors on the wrist may underrepresent total arm activity, particularly during tasks involving the hand and forearm. Only three of the six studies positioned sensors on the wrist to capture activity following RSA [13,15,16]. For example, Edwards et al. [15] included both wrist- and upper arm-worn sensors in patients following RSA and found that sensors placed on the wrist recorded higher mean activity levels at each time point when compared to the sensors placed on the upper limb, probably due to the higher sensitivity of capturing forearm and hand movement. Irrespective of the sensor placement, similar recovery trajectories and the normalization of limb symmetry by 12 months post-RSA were observed in both the wrist and upper arm.

One of the more obvious variations we observed in this review was for wear time and session duration. Most reported wear time protocols that ranged from 8 to 10 h per day to full waking hours. Session length (or the total period of capture time) varied from only a single-day capture for some, to continuous wear time for ≥7 days for others. Three of the six studies employed wear time and capture periods for only a single day which may not reflect the accurate or typical upper limb movement or functional demands of a patient in their free-living environment. Short monitoring periods may overlook meaningful fluctuations in arm use due to pain and/or fatigue and may also fail to account for variation related to routine tasks or scheduled recreational activities. It is for this reason that between 7 and 10 days of capture has been recommended to ensure the reliable measurement of activity [20]. Future studies that look to implement sensors post-RSA with a minimum of a 7-day period of data capture.

We found in this review that studies either measured shoulder motion or shoulder activity as a measure of recovery following RSA. One of the advantages of RSA is the mechanical advantage it provides to the deltoid to achieve better restoration of shoulder elevation post-surgery, making it an obvious measure of recovery. Previous studies have observed that daily arm movements are predominantly performed below 90° of elevation [21], with more than 95% of activity occurring within this range both before and after RSA [18]. Van de Kleut et al. [18] reported that 95% of movement time 12 months post-RSA occurs in ranges below 60° of elevation and only 1% of movement time above 90° of elevation. This has been observed in other studies which show that even as the range of motion of the arm improves postoperatively, patients do not appear to utilize the greater capacity of shoulder elevation in daily life. This probably reflects the fact that most activities of daily living rarely require elevation beyond 100° [17]. This suggests that metrics based on shoulder elevation-based metrics alone may not adequately capture a complete picture of recovery following RSA and that consideration should be given to activity-based metrics.

Beyond measuring shoulder elevation, evaluating how patients use their shoulder during daily activities provides important insight into functional recovery based on the premise that an increase in arm activity following RSA may reflect improved confidence and capacity to use the shoulder post-surgery, while a reduction in use, or activity avoidance, may signal delayed or incomplete recovery [15]. Quantifying both the volume and intensity of upper limb activity has been studied following RSA and rotator cuff tears [15,16,18,19]. Van der Kleut et al. [18] for example assessed daily arm activity by converting accelerometer data into 1 min epochs, categorizing them into low-, moderate-, or high-intensity counts. Despite improvements in clinical ROM, shoulder activity intensity did not change from baseline to 3 or 12 months, with most of the day spent on low- to moderate-intensity activity. Hurd et al. [16] reported upper arm movement over a period of three days, at 2 and 12 months post-surgery. They reported movement as activity counts, quantified using the vector magnitude of triaxial accelerations per epoch, and dichotomized activity counts into ‘activity bins’ to reflect the time spent inactive and the time spent engaged in movement of low- and/or high-frequency. No changes in inactivity or low-frequency activity between pre- and post-surgery time points were observed, and although high-frequency activity increased from 2 to 12 months post-surgery, it remained similar to preoperative levels.

Wrist-worn wearable sensors in the form of smartwatches and fitness trackers have grown in popularity over the past decade. In orthopedics, this has enabled the development of large-scale remote monitoring platforms such as Zimmer Biomet’s mymobility^®^, which uses Apple Watches to record step counts and gait metrics following lower limb arthroplasty [9,10,22,23]. To our knowledge, this sort of monitoring has not been employed for the shoulder, where the functional focus is on shoulder elevation or activity, rather than gait. Some total knee arthroplasty implants now incorporate embedded IMUs capable of recording gait metrics data with a battery life exceeding 10 years [24]. No comparable technology exists for RSA, and unlike the knee, the shoulder lacks both suitable implant design and the consistent, predictable cyclic loading of gait, making embedded IMU monitoring difficult. However, in one of the studies included in this scoping review, daily shoulder elevation cycles may serve as an upper limb equivalent to gait metrics and step counts as observed in lower limb arthroplasty [9,10,13]. Langohr et al. [13] reported that patients following RSA completed an average of 809 and 822 humeral elevation movements per hour on the operated and nonoperated arms, respectively, with most of these movements occurring below 80° of elevation. When extrapolated, this equates to 0.75 million elevation cycles above 60° per year, a volume suggested to be comparable to lower limb joint use [9,10]. This measure may be used as an indication to inform both functional recovery and implant wear modeling, much like step counts are used in lower limb arthroplasty monitoring. Future studies should explore this.

None of the six studies used the activity or motion metrics to guide rehabilitation decision-making or exercise compliance. Previous research has shown that activity counts from wrist-worn accelerometers strongly correlate with visually observed arm movements during activities of daily living [25]. This supports the possible utility of the remote monitoring of exercise and activity compliance during early rehabilitation following RSA. Using activity counts in a similar way to how they have been used in lower limb arthroplasty, trajectories in the recovery of shoulder function and activity could provide clinicians with the ability to better understand the improvement of shoulder function by comparing to benchmarks, to guide rehabilitation progression, reduce unnecessary follow-up visits, and identify patients exhibiting atypical recovery who may benefit from intervention to prevent adverse outcomes.

This review has limitations. In particular, the small number of studies and the variation in their methods and measurements, as well as small sample sizes, meant that our ability to make direct comparisons between studies was limited. Short follow-up durations and the absence of preoperative data in most studies further limited the assessment of recovery over time. Finally, as wearable sensor technologies continue to evolve, it is likely that new studies and new methods have emerged since the completion of our search.

## 5. Conclusions

This scoping review found that the application of wearable sensors after RSA was highly variable, in particular, the sensor, placement, sampling frequency, wear time, and session duration. While studies clearly sought to measure either activity or arm motion, the specific outcome metrics used to describe either of these were also variable. Such heterogeneity limits the ability to compare findings across studies and makes it difficult to make consistent conclusions. These results highlight the need for greater standardization in sensor protocols and outcomes to enable the reliable assessment of postoperative recovery after RSA.

## Figures and Tables

**Table 1:** Search terms included in the MEDLINE database.

#	Search Terms
1	Arthroplasty, Replacement, Shoulder/
2	(shoulder arthroplast* OR shoulder replace*).mp
3	Wearable electronic device/
4	Wearable*.ti,ab.
5	(IMU* OR inertial measurement unit* OR inertial sens*).mp
6	(smart watch* OR fitbit* OR apple watch* OR activity monitor* OR activity tracker* OR garmin* OR activpal OR pedometer OR actigraph*).mp
7	(accelerometer* OR gyroscope*).mp
8	1 OR 2
9	3 OR 4 OR 5 OR 6 OR 7
10	8 AND 9

**Table 2:** Study characteristics.

Study	Year	Study Design	Sample Size	Sample Demographics	Follow-Up Time Points	Setting	Implant Type(s)
Chapman et al. [17]	2023	Prospective pilot study	RSA (n = 10); control (n = 10)	RSA: Mean age: 82.0 y ± 5.0; 1 M/9 F Control: Mean age: 69.0 y ± 20.0; 4 M/6 F	Preoperative, 3, 12, 24 months	Free-living	Trabecular Metal Reverse Shoulder System (Zimmer Biomet)
Edwards et al. [15]	2020	Prospective non-randomized	RSA (n = 36)	Mean age: 73.9 y (range: 56–84); 61% F	3, 6 and 12 months	Free-living	Equinoxe Reverse Shoulder Design (Exactech)
Hurd et al. [16]	2018	Prospective cohort study	RSA (n = 14)	Mean age 73.0 y ± 6.0; 7 M/7 F	Preoperative, 2 and 12 months	Free-living	Not reported
Langohr et al. [13]	2018	Basic science study	RSA (n = 20); aTSA (n = 16)	Mean age 73.0 y ± 10.0 *^,†^	>12 months	Free-living	Not specified
Morgan et al. [14]	2024	Prospective cohort study	RSA (n = 28); aTSA (n = 36)	Mean age: 68.7 y (range: 38–86); 60% M *	Preoperative and 24 weeks	Free-living	Biomet (n = 40), Tornier Flex (n = 21) and Simpliciti (n = 2)
Van de Kleut et al. [18]	2021	Prospective case series	RSA (n = 33)	Mean age 71.8 y ± 8.0; 58% M	Preoperative, 3 and 12 months	Free-living	Aequalis Ascend Flex (Wright Medical-Tornier Group

* Breakdown of demographics by group not reported; ^†^ sex details not included. aTSA, anatomic total shoulder arthroplasty; F, female; M, male; RSA, reverse shoulder arthroplasty; y, years.

**Table 3:** Summary of wearable sensor protocols in RSA studies, including sensor placement protocols, wear time, and session duration.

Study	Sensor Type	Axes	Components Used	Sampling Rate	Sensor Placement	No. of Sensors	Session Length	Wear Time
Chapman et al. [17]	APDM IMU	3	Accelerometer, gyroscope, magnetometer	Not specified	Unilateral humerus (operated limb only), and sternum	2	>7 days	>8 h/day
Edwards et al. [15]	ActiGraph GT9X Link	3	Accelerometer	100 Hz	Bilateral humeri, bilateral wrists	4	≤3 days	≥10 h/day
Hurd et al. [16]	ActiGraph GT3X+	3	Accelerometer	100 Hz	Unilateral humerus and wrist (operated limb only)	2	≤3 days	≥10 h/day
Langohr et al. [13]	YEI Technology IMU	3	Accelerometer, gyroscope, compass	Not specified	Sternum, bilateral humeri, bilateral wrists via compression shirts	5	<24 h	Full waking hours
Morgan et al. [14]	ActiGraph GT9X Link	3	Accelerometer, gyroscope, magnetometer	100 Hz	Unilateral humerus (operated limb only)	1	<24 h	Full waking hours
Van de Kleut et al. [18]	3-Space Data Logger; Yost Labs	3	Accelerometer, gyroscope, compass	10 Hz	Unilateral humerus (operated limb only), and sternum	2	<24 h	Full waking hours

**Table 4:** Summary of wearable sensor metrics in RSA studies, including the outcome category, and outcome variables and metrics reported.

Study	Outcome Category	Outcome Variables and Metrics Reported
Chapman et al. [17]	Motion	Average degrees elevation (weekly); maximum degrees elevation (weekly); per cent time in 15° bins <90°; per cent time in 45° bins over >90°.
Edwards et al. [15]	Activity	Mean activity values (calculated from the vector magnitude per 15 s epoch), limb symmetry index, magnitude ratio.
Hurd et al. [16]	Activity	Mean activity values (calculated from the vector magnitude per 60 s epoch), per cent time inactive or in low-intensity or high-intensity activity.
Langohr et al. [13]	Motion	Per cent time spent in elevation ranges (e.g., <60°, 60–80°, >80°, >100°); per cent time in elevation planes (forward flexion = 0°); motion frequency (e.g., elevations) per hour; estimated annual cycles of the shoulder extrapolated from daily data.
Morgan et al. [14]	Activity	Mean activity count (counts/sec) per axis; mean vector magnitude per 1 s epoch; per cent time spent in sedentary, light, moderate, vigorous, and very vigorous activity.
Van de Kleut et al. [18]	Motion	Elevation events per hour; elevation events per hour under >90°; per cent time spent >90° elevation; per cent time spent in low-, moderate-, and high-intensity activity per day.

## Data Availability

Not applicable.

## References

[1] Smith P.N., Gill D.R., McAuliffe M.J., McDougall C., Stoney J.D., Vertullo C.J., Wall C.J., Corfield S., Page R., Cuthbert A.R. (2023). Hip, Knee and Shoulder Arthroplasty: 2023 Annual Report.

[2] Garcia G.H., Taylor S.A., DePalma B.J., Mahony G.T., Grawe B.M., Nguyen J., Dines J.S., Dines D.M., Warren R.F., Craig E.V. (2015). Patient Activity Levels After Reverse Total Shoulder Arthroplasty: What Are Patients Doing?. Am. J. Sports Med..

[3] MacInnes S.J., Mackie K.E., Titchener A., Gibbons R., Wang A.W. (2019). Activity following reverse total shoulder arthroplasty: What should surgeons be advising?. Shoulder Elb..

[4] Simovitch R.W., Gerard B.K., Brees J.A., Fullick R., Kearse J.C. (2015). Outcomes of reverse total shoulder arthroplasty in a senior athletic population. J. Shoulder Elb. Surg..

[5] Roche C., Kumar V., Overman S., Simovitch R., Flurin P.-H., Wright T., Routman H., Teredesai A., Zuckerman J. (2021). Validation of a machine learning–derived clinical metric to quantify outcomes after total shoulder arthroplasty. J. Shoulder Elb. Surg..

[6] Schoch B.S., King J.J., Fan W., Flurin P.-H., Wright T.W., Zuckerman J.D., Roche C.P. (2022). Characteristics of anatomic and reverse total shoulder arthroplasty patients who achieve ceiling scores with 3 common patient-reported outcome measures. J. Shoulder Elb. Surg..

[7] Eckhard L., Munir S., Wood D., Talbot S., Brighton R., Walter B., Baré J. (2021). The ceiling effects of patient reported outcome measures for total knee arthroplasty. Orthop. Traumatol. Surg. Res..

[8] Small S.R., Bullock G.S., Khalid S., Barker K., Trivella M., Price A.J. (2019). Current clinical utilisation of wearable motion sensors for the assessment of outcome following knee arthroplasty: A scoping review. BMJ Open.

[9] Christensen J.C., Blackburn B.E., Anderson L.A., Gililland J.M., Peters C.L., Archibeck M.J., Pelt C.E. (2023). Recovery Curve for Patient Reported Outcomes and Objective Physical Activity After Primary Total Knee Arthroplasty-A Multicenter Study Using Wearable Technology. J. Arthroplast..

[10] Christensen J.C., Blackburn B.E., Kapron C.R., Pelt C.E., Peters C.L., Archibeck M.J., Anderson L.A., Gililland J.M. (2025). Unicompartmental Knee Arthroplasty Patients Recover More like Total Hip Patients than Total Knee Patients: A Prospective Longitudinal Study. J. Arthroplast..

[11] The Joanna Briggs Institute The Joanna Briggs Institute Reviewer’s Manual: Methodology for JBI Scoping Reviews. https://www.joannabriggs.org.

[12] Tricco A.C., Lillie E., Zarin W., O’Brien K.K., Colquhoun H., Levac D., Moher D., Peters M.D.J., Horsley T., Weeks L. (2018). PRISMA Extension for Scoping Reviews (PRISMA-ScR): Checklist and Explanation. Ann. Intern. Med..

[13] Langohr G.D.G., Haverstock J.P., Johnson J.A., Athwal G.S. (2018). Comparing daily shoulder motion and frequency after anatomic and reverse shoulder arthroplasty. J. Shoulder Elb. Surg..

[14] Morgan C., Hargreaves M., Williams M., Hoyt R.E., Snider D.H., Callanan M., Nelson A., Brabston E.W., Momaya A.M., Ponce B.A. (2024). The use of actigraphy to objectively define motion and function before and after shoulder arthroplasty. J. Orthop..

[15] Edwards P.K., Ebert J.R., Morrow M.M., Goodwin B.M., Ackland T., Wang A. (2020). Accelerometry evaluation of shoulder movement and its association with patient-reported and clinical outcomes following reverse total shoulder arthroplasty. J. Shoulder Elb. Surg..

[16] Hurd W.J., Morrow M.M., Miller E.J., Adams R.A., Sperling J.W., Kaufman K.R. (2018). Patient-Reported and Objectively Measured Function Before and After Reverse Shoulder Arthroplasty. J. Geriatr. Phys. Ther..

[17] Chapman R.M., Torchia M.T., Bell J.-E., Van Citters D.W. (2023). Using inertial measurement units to quantify shoulder elevation after reverse total shoulder arthroplasty: A pilot study comparing goniometric measures captured clinically to inertial measures captured ‘in-the-wild’. Semin. Arthroplast. JSES.

[18] Van de Kleut M.L., Bloomfield R.A., Teeter M.G., Athwal G.S. (2021). Monitoring daily shoulder activity before and after reverse total shoulder arthroplasty using inertial measurement units. J. Shoulder Elb. Surg..

[19] Hurd W.J., Morrow M.M., Kaufman K.R. (2013). Tri-axial accelerometer analysis techniques for evaluating functional use of the extremities. J. Electromyogr. Kinesiol..

[20] Hilden P., Schwartz J.E., Pascual C., Diaz K.M., Goldsmith J. (2023). How many days are needed? Measurement reliability of wearable device data to assess physical activity. PLoS ONE.

[21] Coley B., Jolles B.M., Farron A., Aminian K. (2008). Arm position during daily activity. Gait Posture.

[22] Fary C., Cholewa J., Abshagen S., Van Andel D., Ren A., Anderson M.B., Tripuraneni K. (2023). Stepping Beyond Counts in Recovery of Total Hip Arthroplasty: A Prospective Study on Passively Collected Gait Metrics. Sensors.

[23] Fary C., Cholewa J., Abshagen S., Van Andel D., Ren A., Anderson M.B., Tripuraneni K.R. (2023). Stepping beyond Counts in Recovery of Total Knee Arthroplasty: A Prospective Study on Passively Collected Gait Metrics. Sensors.

[24] Yocum D., Elashoff B., Verta P., Armock G., Yergler J. (2023). Patient reported outcomes do not correlate to functional knee recovery and range of motion in total knee arthroplasty. J. Orthop..

[25] Lawinger E., Uhl T.L., Abel M., Kamineni S. (2015). Assessment of Accelerometers for Measuring Upper-Extremity Physical Activity. J. Sport Rehabil..

